# Blood levels of D-aspartate oxidase, D-amino acid oxidase, serine racemase, and pLG72 are influenced by diagnoses of schizophrenia and autism spectrum disorder

**DOI:** 10.1038/s41537-026-00758-7

**Published:** 2026-04-25

**Authors:** Elisa Maffioli, Francesco Errico, Zoraide Motta, Raffaella di Vito, Joshua Grana, Elisa De Grandis, Silvia Boeri, Claudio Bruno, Maria Pia Riccio, Felice Iasevoli, Michele Di Maio, Tommaso Nuzzo, Carmela Bravaccio, Sveva Bagnasco, Monica Gelzo, Giuseppe Castaldo, Andrea de Bartolomeis, Armando Negri, Loredano Pollegioni, Gabriella Tedeschi, Alessandro Usiello

**Affiliations:** 1 DIVAS, Department of Veterinary Medicine and Animal Sciences, University of Milano, Milan, Italy; 2 Department of Agricultural Science, University of Naples “Federico II”, Portici, Italy; 3 CEINGE Biotecnologie Avanzate “Franco Salvatore”, Naples, Italy; 4 “The Protein Factory 2.0”, Dipartimento di Biotecnologie e Scienze della Vita, Università degli Studi dell’Insubria, Varese, Italy; 5 Department of Environmental, Biological and Pharmaceutical Sciences and Technologies, Università degli Studi della Campania “Luigi Vanvitelli”, Caserta, Italy; 6 Department of Neuroscience, Rehabilitation, Ophthalmology, Genetics, Maternal, and Child Health – DINOGMI, University of Genoa, Genoa, Italy; 7 Child Neuropsychiatry Unit, IRCCS Istituto Giannina Gaslini, Genoa, Italy; 8 Center of Translational and Experimental Myology, Istituto di Ricovero e Cura a Carattere Scientifico (IRCCS) Istituto Giannina Gaslini, Genoa, Italy; 9 Department of Maternal and Child Health, Unità Operativa semplice di Dipartimento (UOSD) of Child and Adolescent Psychiatry, Azienda Ospedaliera Universitaria (AOU) Federico II, Naples, Italy; 10 Section of Psychiatry, Laboratory of Translational and Molecular Psychiatry and Unit of Treatment-Resistant Psychosis, Department of Neuroscience, Reproductive Sciences and Odontostomatology, University Medical School of Naples “Federico II”, Naples, Italy; 11 Department of Medical and Translational Sciences, Child Neuropsychiatry, Federico II University, Napoli, Italy; 12 Dipartimento di Medicina di Precisione in Area Medica, Chirurgica e Critica, Università di Palermo, Palermo, Italy; 13 Department of Molecular Medicine and Medical Biotechnologies, Federico II University of Naples, Naples, Italy

**Keywords:** Schizophrenia, Schizophrenia

## Abstract

Free D-serine (D-Ser) and D-aspartate (D-Asp) are increasingly recognized as key modulators of glutamatergic NMDA receptor-dependent neurotransmission, whose dysfunction has been implicated in neuropsychiatric conditions, including schizophrenia (SCZ) and autism spectrum disorder (ASD). The metabolism of these D-amino acids is tightly regulated by specific enzymes: serine racemase (SR) for D-Ser synthesis and degradation, and D-amino acid oxidase (DAAO) and D-aspartate oxidase (DASPO) for D-Ser and D-Asp degradation, respectively. The primate-specific protein pLG72 further modulates the activity of DAAO and DASPO. In this multicenter study, we employed a mass spectrometry (MS)-based approach to quantify SR, DAAO, DASPO, and pLG72 levels in serum samples from SCZ and ASD patients, along with matched non-psychiatric controls. Enzymatic activity and D-amino acid serum concentrations were also assessed. We identified distinct, disorder-specific alterations in these proteins. In SCZ patients, SR protein levels were elevated despite unchanged activity, while DAAO and pLG72 levels were decreased. Conversely, increased DASPO levels were associated with reduced D-Asp, indicating enhanced catabolism of this endogenous NMDA receptor ligand in SCZ. ASD patients exhibited elevated DAAO and DASPO, with reduced SR levels. Notably, positive correlations between pLG72 and both DAAO and DASPO flavoenzymes were observed in both disorders. These findings highlight the potential of D-amino acid metabolism-related enzymes as biomarkers for SCZ and ASD and provide new insights for future diagnostic and mechanistic investigations in neurodevelopmental disorders.

## Introduction

Amino acids are the building blocks of proteins and exist in two mirror-image forms: L- and D-enantiomers. While L-amino acids are predominant in most biological systems and central to protein synthesis, D-amino acids (D-AAs) are now recognized as important modulators of neural functions^1,2^. Among these, D-serine (D-Ser) and D-aspartate (D-Asp) have garnered particular attention for their roles in the central nervous system (CNS) and psychiatric diseases, above all schizophrenia (SCZ) and autism spectrum disorder (ASD)^3^, both conceptualized as neurodevelopmental and synaptic plasticity disorders^4,5^.

D-Ser is synthesized endogenously from the L-enantiomer through the action of the enzyme serine racemase (SR, EC 5.1.1.18) in neurons^6–10^. In the CNS, D-Ser is mainly degraded by the flavin adenine dinucleotide-dependent peroxisomal enzyme D-amino acid oxidase (DAAO, EC 1.4.3.3), which catalyzes its conversion into hydroxypyruvate, ammonia, and hydrogen peroxide^11–13^. D-Ser plays a crucial role in modulating neurotransmission within the CNS since it functions as a potent co-agonist at the glycine-binding site of N-methyl-D-aspartate receptors (NMDARs)^14^. Therefore, D-Ser is now recognized as a fundamental modulator of central excitatory neurotransmission, playing a key role in synaptic plasticity and higher cognitive functions^1^. In SCZ, reduced serum and brain contents of D-Ser have been reported in multiple cohorts of patients^9^, and are associated with executive function performance^15,16^, whereas elevated levels, either through direct supplementation or inhibition of DAAO enzyme, correlate with improvements in pre-attentive and cognitive functions^17–19^.

D-Asp is degraded by the flavoenzyme D-aspartate oxidase (DASPO or DDO, EC 1.4.3.1)^20,21^. DASPO is essential for maintaining physiological levels of D-Asp within the CNS, as it is the sole catabolic enzyme acting on free D-Asp in mammals^20,22^. To date, a specific enzyme for D-Asp biosynthesis has not been identified in mammals. However, some studies suggest that SR may also contribute to D-Asp synthesis^21,23^. This D-AA also plays a critical role in neurotransmission, by binding to the glutamate-binding site on the GluN2 subunits of NMDARs, and to metabotropic glutamate receptor 5 (mGluR5)^24,25^. Based on its modulatory role, several studies have shown that D-Asp contributes to NMDAR-dependent synaptic plasticity, brain activity, dendritic growth and spine density^26–33^. Specifically, D-Asp levels are particularly elevated during brain development, decreasing significantly after birth due to the postnatal activity of DASPO^34–36^. This peculiar ontogenetic trajectory suggests a pivotal role for D-Asp in neurodevelopmental processes and supports the hypothesis that altered metabolism of this NMDAR ligand may contribute to the pathogenesis of neuropsychiatric conditions, including SCZ and ASD^37–41^. In line with this notion, post-mortem brain investigations have revealed significantly reduced D-Asp levels in individuals with SCZ, associated with increased DASPO mRNA expression or enzymatic activity in the prefrontal cortex^37,39^. Moreover, alterations in cortical D-Asp metabolism, along with D-Ser levels, have been proposed as a potential discriminative index between SCZ and non-psychiatric control conditions in a machine learning study^42^. Additional clinical studies reported selective reduction in serum D-Asp (and D-Ser) levels in patients with fully manifested SCZ, compared to non-psychiatric controls^41^. On the other hand, transient increases in the serum levels of these D-AAs have been observed during the prodromal stages of psychosis, prior to the onset of full-blown SCZ^40^. Consistent with a role for D-Asp dysregulation across neurodevelopmental disorders, increased D-Asp levels have been found in brain regions of the idiopathic ASD mouse model, BTBR^43^, as well as of environmental ASD rat models prenatally exposed to lipopolysaccharide or valproate^44^.

Intracellular levels of D-Ser and D-Asp are tightly controlled by their respective degradative enzymes, DAAO and DASPO, which show high sequence identity and overall tertiary structure, and share the same chemical mechanism of catalysis^13,20,22,45,46^. In recent years, we demonstrated that both flavooxidases interact with the primate-specific protein pLG72, whose Arg30Lys variant was related to SCZ susceptibility^11,47,48^. pLG72 acts as a negative chaperone by inactivating the enzymes and pushing their cellular turnover^46,49–51^, thus preventing excessive degradation of D-Ser and D-Asp.

Based on the key role of DASPO, DAAO, SR and pLG72 in regulating the endogenous D-Asp and D-Ser metabolism, in this study, we used a mass spectrometry (MS)-based approach for the detection and quantification of these proteins in the serum of patients with SCZ or ASD and their relative non-psychiatric controls. Moreover, we combined MS analysis with enzymatic assays and used enantiomeric HPLC detections to correlate serum enzyme expression with D-AA levels. Overall, our multi-approach analysis revealed significant enzyme alterations in SCZ and ASD conditions, thus supporting the relevance of proteins related to D-AA metabolism as a circulating biomarker for neuropsychiatric disorders.

## Methods

### Demographic characteristics of healthy subjects

To investigate if serum levels of DASPO, DAAO, SR, and pLG72, as well as D-Asp and D-Ser, differ between males and females, we recruited non-psychiatric adult subjects (*n* = 31) at the “Federico II” University Hospital of Naples. Inclusion criteria were: age 18–60 years, no history of neurological, psychiatric, or systemic conditions or family psychiatric history. Demographic characteristics of healthy subjects are reported in Suppl. Table 1.

### Demographic and clinical characteristics of patients with schizophrenia

Blood serum samples were obtained from SCZ patients (*n* = 25) and non-psychiatric controls (*n* = 12). Patients with schizophrenia were recruited at the “Federico II” University Hospital of Naples over 6 months and diagnosed according to the Diagnostic and Statistical Manual of mental disorders, Fifth Edition (DSM-5)^52^. Inclusion criteria for patients were: age 18–60 years; no evidence of worsening psychotic symptoms in the previous 6 months; absence of other major systemic, psychiatric (e.g., addictive disorders, frequent substance use in the 6 months prior to enrolment, etc.), or neurological disorders. Healthy controls were sex-matched individuals with no history of neurological, psychiatric, or systemic conditions or family psychiatric history. SCZ patients were divided into two groups: non-treatment-resistant (nTRS; *n* = 12) and treatment-resistant (TRS; *n* = 13). The treatment resistance condition was defined according to the modified Treatment Response and Resistance in Psychosis Working Group Consensus criteria as a failure (i.e., less than 25% score reduction at Positive and Negative Symptoms Scale, PANSS) of at least two different antipsychotic treatments, each administered for > 6 weeks and at an optimal dose^53^. All TRS patients were under treatment with clozapine while nTRS patients were treated with different conventional antipsychotics, such as olanzapine, risperidone, haloperidol, amisulpride, promazine, paliperidone and aripiprazole. Clinical data were collected within 1 month from the blood sample and included the severity of psychotic symptoms measured by the PANSS^54^ and cognitive performances assessed by the Brief Assessment of Cognition in Schizophrenia (BACS)^55^. Demographic characteristics are reported in Table 1. Blood collection was conducted in the morning between 8:00 AM and 10:00 AM. Serum was separated by centrifugation and stored at −80 °C until analysis. Written informed consent was obtained from all subjects, according to the Declaration of Helsinki. The study was approved by the Ethics Committee of the University “Federico II” of Naples (protocol number: 195/19).

### Demographic and clinical characteristics of patients with autism spectrum disorder

Blood serum samples were obtained from two different Italian hospitals: “Federico II” University Hospital, Naples, Italy (ASD, *n* = 21; Control, *n* = 5) and Istituto Giannina Gaslini, Genoa, Italy (ASD, *n* = 14; Control, *n* = 12). Participants from “Federico II” University Hospital were consecutive samples of children and adolescents, along 6 months, referred to the Department of Pediatrics — Unit of Child and Adolescent Neuropsychiatry, for an evaluation in a clinical hypothesis or revaluation of ASD. Study participants included 21 ASD subjects. Inclusion criteria were a clinical diagnosis of ASD, less than 18 years of age; exclusion criteria included: epilepsy diagnosis or other neurological disorders; psychiatric comorbidity (e.g. obsessive-compulsive disorder, psychosis, etc.), other chronic diseases (e.g. chronic intestinal diseases, malabsorption, etc.). Five healthy subjects with no history and clinical evidence of development disorders were recruited as a control group; inclusion criteria were the absence of psychiatric diagnosis, less than 18 years of age. For the control group, the same exclusion criteria were used. The enrolled subjects followed routine clinical procedures for outpatients, from which data were collected. Each patient was also investigated by blood samples, as per routine procedures during clinical evaluation. Participants from Istituto Giannina Gaslini were consecutive samples of children and adolescents, referred to the Child Neuropsychiatry Unit Day Hospital for a third-level neuroradiological, biochemical, metabolic and genetic evaluation in a clinical diagnosis of ASD. Study participants included 14 ASD subjects aged between 3 years and 6 months and 11 years and 4 months, 13 males and 1 female. Inclusion criteria were a clinical diagnosis of ASD, less than 18 years of age; while exclusion criteria were the presence of other psychiatric diagnosis, epilepsy or other chronic diseases. Twelve developing normal children were recruited as a control group; inclusion criteria were the absence of psychiatric diagnosis, less than 18 years of age. For the control group, the same exclusion criteria were used. For both cohorts, all the subjects received a full assessment, including a complete history (pregnancy, childbirth, psychomotor development), structured clinical interviews and validated^41^. Diagnosis of ASD was formulated according to DSM-5^52^. Blood collection was made in fasting status, in the morning. Serum was separated by centrifugation and stored at −80 °C until analysis. The study was conducted according to the principles of the Declaration of Helsinki; ethical approval was obtained by the Ethics Committee of the University Federico II of Naples (220/18) and the Ethics Committee of the Liguria Region (N. CET – Liguria: 437/2023 – DB id 13411). Written informed consent was collected from parents or legal guardians of enrolled children for both clinical information collection and data acquisition and treatment. Due to the low number of control subjects from the “Federico II” University Hospital, the two cohorts were merged for the analysis. Demographic characteristics of ASD and control individuals from the unified cohort, such as age and sex distribution, are reported in Table 1.

### D-amino acid HPLC analysis

We quantified D-Asp and D-Ser levels in the blood serum by HPLC as previously described^41,56^ with minor modifications. Identification and quantification of D-Asp and D-Ser were based on retention times and peak areas, compared with that associated with external standards. The identity of the peaks was further confirmed either with internal standards and the selective degradation by RgDAAO M213R variant^57^. The concentration of D-amino acids in the serum was expressed as micromolar (µM).

### Sample preparation for MS analysis

Recombinant human DAAO, DASPO and pLG72 proteins were expressed in *E. coli* and purified according to^46,58,59^. Human SR was a generous gift of Barbara Campanini, University of Parma. Protein and human serum samples were denatured in 8 M urea, 20 mM HEPES, pH 8.0^60^ reduced with 13 mM DTT, alkylated with 26 mM iodoacetamide and digested overnight with trypsin (Promega) at 37 °C, protein: enzyme ratio 20:1. Peptides were desalted (Zip-Tip C18), dried and reconstituted in 0.1% formic acid (FA)^60^. For PRM analysis, bovine cytochrome c derived-peptides (Cyt c, 0.5 pmol, Thermo Fisher) were spiked into digested a internal standard.

### Liquid chromatography–tandem mass spectrometry analysis

**Fig. 1:**
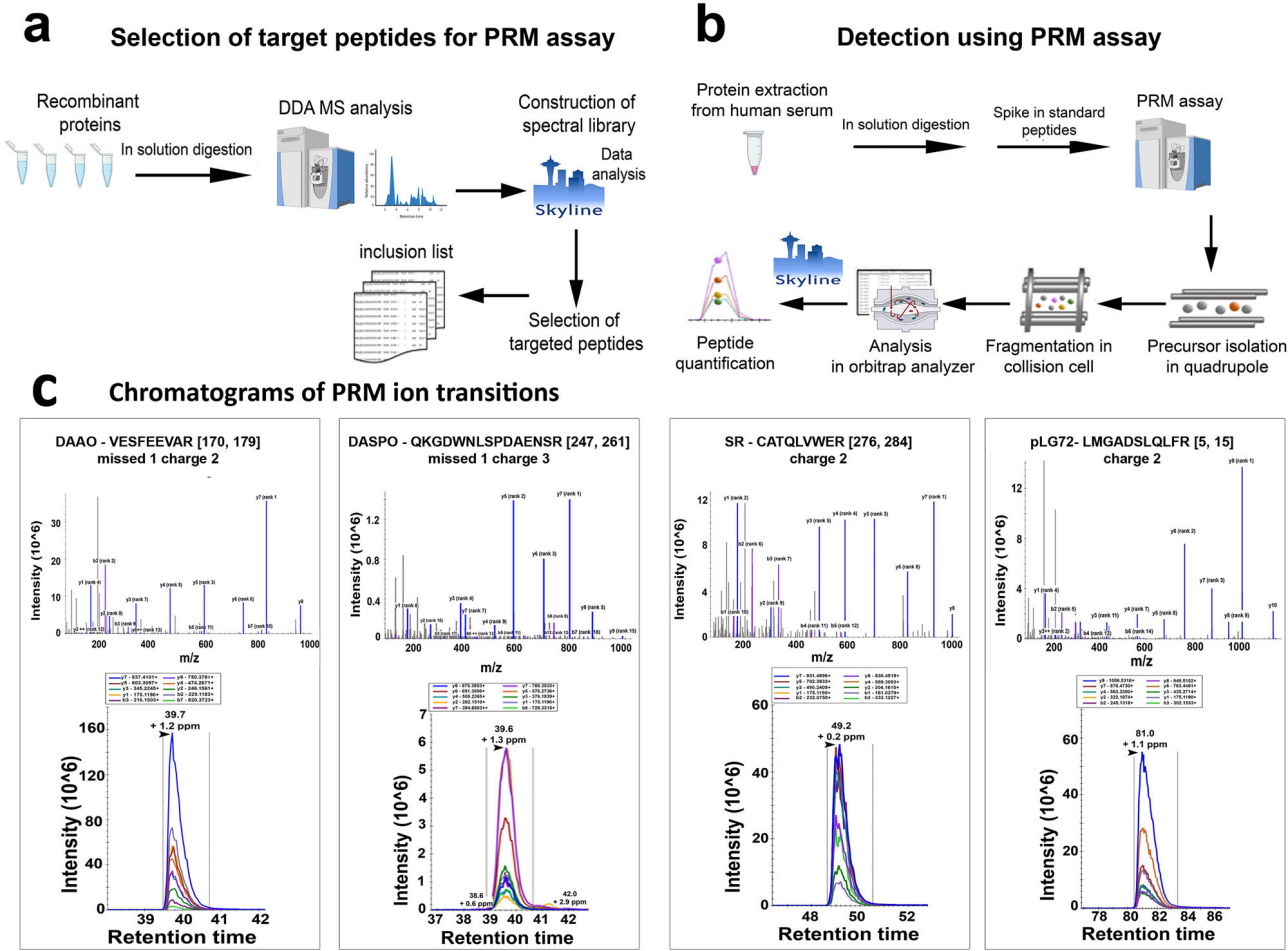
Parallel reaction monitoring (PRM) assay for detection of, DAAO, DASPO, SR and pLG72 in blood serum of control, schizophrenia and autism spectrum disorder patients. **a** Peptide sequences of recombinant proteins (DAAO, DASPO, SR and pLG72) were obtained through data dependent acquisition (DDA) MS analysis. A spectral library for DAAO, DASPO, SR and pLG72 was constructed in Skyline software and used for PRM-based targeted MS quantification of proteins. **b** Overview of the experimental workflow for developing the PRM assay to quantify DAAO, DASPO, SR and pLG72 in blood serum. Target peptides from the inclusion list from all recombinant proteins were isolated and fragmented from the sample on the basis of the mass-to-charge ratio, and all fragments were analyzed in parallel on a high-resolution. **c** Example of MS/MS spectrum and extracted-ion chromatogram (XIC) of the transitions observed for the peptides _170-_VESFEEVAR_-179_, _247-_QKGDWNLSPDAENSR_-261_, _276-_CATQLVWER_-284_ and _5-_LMGADSLQLFR_-15_ derived from DAAO, DASPO, SR and pLG72, respectively, as measured by PRM.

Data-dependent acquisition (DDA) and PRM analyses (Fig. 1 a, b) were carried out on a Q-Exactive Plus Orbitrap mass spectrometer (Thermo Fisher Scientific) interfaced with a Dionex Ultimate 3000 NanoHPLC system with an EASY-SprayTM 2 µm 15 cm × 150 cm capillary column filled with 2 µm C18 100 Å particles. Peptide mixtures were separated using mobile phase A (0.1% formic acid in water) and mobile phase B (0.1% formic acid in acetonitrile 20/80, v/v) at a flow rate of 0.300 µL/min. The LC gradient was: 1% acetonitrile (ACN) in 0.1% formic acid for 10 min, 1–5% ACN in 0.1% formic acid for 6 min, 5–38% ACN in 0.1% formic acid for 147 min and 38–63% ACN in 0.1% formic for 3 min. The temperature was set to 35 °C^61^.

The DDA-mode analytical conditions consisted of a full MS scan at a resolution of 70,000 and a scan range from 375 to 1500 *m/z* (the mass-to-charge ratio) with the automatic gain control (AGC) target value being set to 1 × 10^6^, followed by a data-dependent MS^2^ mode of the 10 most intense peaks from full scan using an isolation window of 3 m/z, AGC 5 × 10^4^ and a normalized collision energy of 35. HCD MS/MS spectra were acquired in Orbitrap at the resolution of 17, 500. Dynamic exclusion was set to 30 s. Rejection of +1 and unassigned charge states were enabled^62^.

In PRM mode, inclusion lists for target peptides (from recombinant proteins and Cyt c) were generated via Skyline (v24.1). MS^2^ scans were acquired at 35,000 resolution, AGC 2×10⁵, max IT 100 ms, isolation window 1.5 m/z. Peptide identification used Skyline with trypsin specificity (max 2 missed cleavages), fixed carbamidomethylation, variable Met oxidation and Asn/Gln deamidation, and FDR ≤ 5%. Quantification was performed against a spectral library built from DASPO or DAAO, or pLG72 or SR DDA-MS data using Skyline (Fig. 1c).

### Activity assays

Serum samples were diluted 1:10 in 20 mM sodium phosphate buffer (pH 8.0) containing 0.1% Triton X-100 (93426, Fluka), supplemented with complete protease- (11836153001, Roche) and phosphatase- (5870, Cell Signaling) inhibitors cocktails. Samples were subsequently centrifuged at 16,000 xg for 30 min at 4 °C. Two µL were used for each reaction for DAAO and DASPO enzymatic activity assays, while 5 µL were utilized for the SR assay.

DAAO and DASPO enzymatic activities were assessed using an Amplex UltraRed-based fluorometric assay to detect H₂O₂ production^61^. The reaction mixture contained 20 µM Amplex UltraRed (Thermo Fisher Scientific), 0.05 U/mL horseradish peroxidase 2.5 mM sodium azide, and FAD (40 µM and 5 µM for DAAO and DASPO reactions, respectively). Substrate concentrations were 100 mM D-Ala for DAAO or 80 mM D-Asp for DASPO. A calibration curve was generated using known amounts of H_2_O_2_ (0.1–10 µM range). Controls without samples or without substrates, as well as reactions containing specific inhibitors (1 mM CBIO for DAAO or 20 mM meso-tartaric acid for DASPO) were assayed simultaneously for validation.

SR activity, analysed for its β-elimination reaction, was measured in a reaction mixture containing 50 mM triethanolamine (TEA), 200 mM L-Ser, 2 mM ATP, 50 µM PLP, 5 mM DTT, 1 mM MgCl₂, 150 mM NaCl, 60 U/mL lactate dehydrogenase (LDH), and 300 µM NADH, pH 8.0; as described by^63^ with minor modification. Control reactions without samples, substrate or LDH were included to validate the assay specificity. A calibration curve was generated using known concentrations of NADH (1–100 µM).

Different amounts of recombinant enzymes were analysed in parallel to enable the quantification of the three target enzymes in serum samples. All enzymatic assays were performed at 25 °C on 96-well plates. The fluorescence intensity at 590 nm (excitation wavelength 535 nm) for the Amplex UltraRed-based assays and the decrease of absorbance intensity at 340 nm were recorded at 30, 60, 120, 240, 360 min and overnight using a microplate reader (Tecan, Infinite M Plex).

### Catalase assay

Serum samples were combined into pools of 5–6 samples each (samples exhibiting possible haemolysis were excluded to minimize interference and ensure accurate catalase quantification). Samples were diluted 1:1500, and catalase levels were measured using a commercial ELISA kit (CSB-E13635h, Cusabio Biotechnology, Wuhan, China) according to the manufacturer’s instructions. Concentrations were calculated from the standard curve; samples were analysed in duplicate; results are expressed as ng/mL.

### Western blot analysis

Peripheral venous blood samples of CTR samples were collected at the Ospedale di Circolo and Fondazione Macchi in Varese (ASST Settelaghi), Italy. Withdrawal of venous blood was performed after a fasting night, between 8:00 and 10:00 a.m., in BD Vacutainer™ SST™ II Advances Tubes (Becton Dickinson, Franklin Lakes, NJ, USA) including clot activator and gel for serum separation. Serum was separated by centrifugation and then stored at -80 °C. Serum sample was treated with HiTrap Albumin & IgG depletion (28947575AD, Cytiva), lyophilized and resuspended in MilliQ. 40 µL of samples were subjected to SDS-PAGE separation and subsequently transferred to a PVDF membrane using the Mini Trans-Blot Cell system (Bio-Rad). Membranes were incubated with specific primary antibodies for 2 h at room temperature: anti-DAAO (Abcam, ab187525; 1:1000) and home-made anti-DASPO (1:1000). The incubation with anti-pLG72 (Invitrogen, PA5-97653; 1:1000) and home-made anti-SR (1:100) antibodies was carried out overnight at 4 °C. As positive controls, and to determine the detection limits of the assay, 25 ng of the corresponding recombinant proteins were added to each sample.

### Statistical analysis

Statistical analyses were performed using SPSS software version 27 (SPSS Inc., Chicago, IL, USA) and Prism 8, version 8.0.2. Normality distribution was assessed using the Kolmogorov-Smirnov and Shapiro–Wilk tests. Quantitative variables were expressed by the median and interquartile range (IQR), while qualitative variables were by absolute frequency. Due to the non-normal data distribution, differences between independent groups were studied by the non-parametric Kruskal-Wallis test, followed by post-hoc Dunn’s test with Bonferroni’s correction or Mann-Whitney test. The effect of confounders was evaluated using ANCOVA model, adjusted for age and/or sex, on natural log-transformed variables. The correlation was evaluated using a non-parametric Spearman test. Groups were considered significantly different when *p* ≤ 0.05.

## Results

In this study, we aimed to analyze DAAO, DASPO, SR and pLG72 proteins in the serum of SCZ and ASD patients, compared to the corresponding non-psychiatric controls. We enrolled patients diagnosed with SCZ (*n* = 25) and controls (Ctrl, *n* = 12) from “Federico II” University Hospital (Italy). SCZ patients were stratified into nTRS (*n* = 12), and TRS (*n* = 13) subgroups. Pediatric ASD patients (*n* = 35) and matched controls (*n* = 17) were enrolled from two additional clinical sites: Istituto Giannina Gaslini and “Federico II” University Hospital (Italy).

### Western blot assessment of serum DAAO, DASPO, SR, and pLG72 protein levels

In an initial approach, we attempted to detect and quantify DAAO, DASPO, SR and pLG72 proteins in the serum of a subset of control individuals using commercial antibodies through Western blot analyses. However, this method failed to yield detectable signals for the endogenous proteins. The detection limit using purified recombinant proteins was 1 ng for DAAO and SR, and 5 ng for DASPO and pLG72 (Suppl. Figure 1), suggesting that the serum concentrations of these targets were below the detection limit by this technique.

Given these limitations, we turned to MS, a more sensitive and specific method capable of detecting low-abundance proteins. However, despite the advantages of MS, proteomic analysis of serum or plasma remains challenging because of the low abundance of target proteins and the wide dynamic range of protein concentrations^64,65^. To overcome these limitations, we developed an MS-based protocol providing very high specificity for the targeted detection and quantification of the key enzymes involved in D-AA metabolism - DAAO, DASPO, SR and the regulatory protein pLG72 (Fig. 1) in serum samples from individuals with SCZ or ASD and their non-psychiatric controls (Table 1, Suppl. Table 1).

**Table 1:** Demographic characteristics of subjects enrolled in the blood serum collection.

Description	Variable	Groups			Statistics	*p*-value
Adult controls and SCZ patients Federico II University Hospital (Figure 2; Suppl. Table 4–7)	Age, median of years [IQR] Sex (M:F)	Controls (*n* = 12) 36 [28-43] 6:6	nTRS (*n* = 12) 50 [37-57] 9:3	TRS (*n* = 13) 34 [29-39] 11:2	F(2,37) = 5.96 χ^2^(2, *n* = 37) = 3.769	0.051^a^ 0.152^b^
Pediatric controls and ASD patients (Figure 3; Suppl. Table 8–10)	Age, median of years [IQR] Sex (M:F)	Controls (*n* = 17) 13.8 [8.6-15.3] 13:4	ASD (*n* = 35) 5.4 [3.9-9.1] 30:5		U(1,51) = 100.50 χ^2^(1, *n* = 52) = 0.683	<0.001^c^ 0.409^b^

Values are expressed as median (IQR) for age. For sex, number of subject (n) is indicated. Statistical analyses were performed by ^a^Kruskall Wallis, ^b^Chi-square test, or ^c^Mann-Whitney tests.

### Serum levels of DAAO, DASPO, SR and pLG72 proteins, and D-serine and D-aspartate are not influenced by sex in control subjects

Before analyzing DAAO, DASPO, SR and pLG72 protein levels in our patient cohorts and corresponding control groups, we evaluated whether sex influences the serum levels of DAAO, DASPO, SR and pLG72, as well as of D-Ser and D-Asp in an independent cohort of adult control subjects enrolled from “Federico II” University Hospital (males, *n* = 20; females, *n* = 11, Suppl. Table 1).

Mann-Whitney analysis on MS-derived data revealed comparable relative serum levels of DAAO, DASPO, SR and pLG72 proteins between male and female control subjects (Suppl. Figure 2a, b, c, d; Suppl. Table 2 and 3). Moreover, enzymatic SR activity did not differ between sexes (Suppl. Figure 2e). Similarly, HPLC analysis showed that the serum content of both D-Asp and D-Ser was not significantly different between sexes (Suppl. Figure 2f, g; Suppl. Table 3).

Then, we assessed the correlation between serum DAAO, SR or pLG72 protein and free D-Ser levels, as well as DASPO, SR or pLG72 protein and free D-Asp levels in the same cohort of control subjects. Non-parametric Spearman analysis did not evidence any significant correlation between protein levels and their respective substrates in either males or females (Suppl. Figure 2h–m; Suppl. Table 3).

Overall, these findings indicated that the serum levels of the investigated D-AAs and their related enzymes/proteins are not significantly affected by sex in control subjects, this strengthening the interpretability of the subsequent comparisons involving patient groups, which are often not balanced for sex distribution (see below).

### The serum levels of DAAO, DASPO, SR and pLG72 are altered in TRS and nTRS schizophrenia patients

**Fig. 2:**
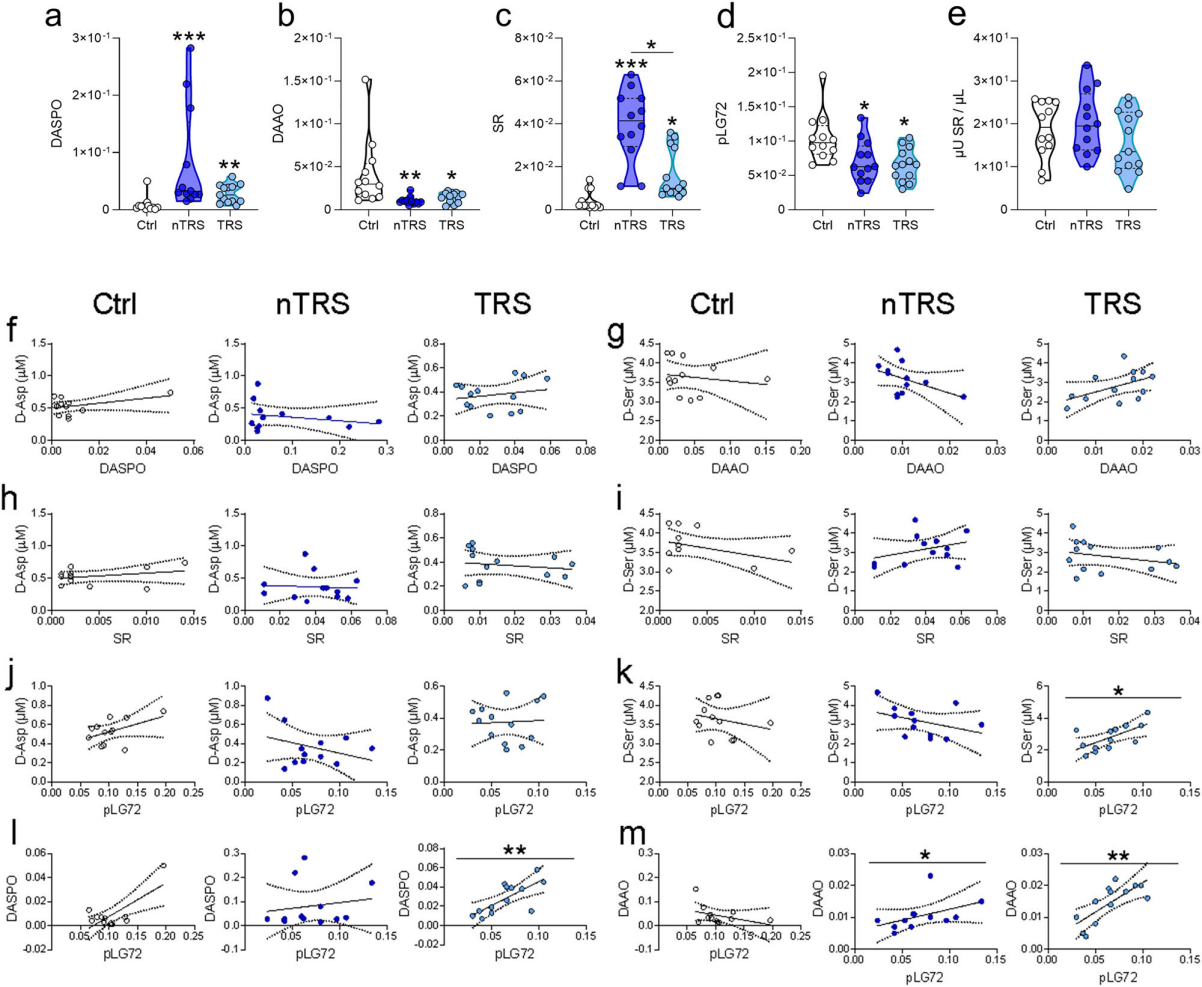
DAAO, DASPO, SR and pLG72 are dysregulated in the serum of patients with schizophrenia. **a** D-aspartate oxidase (DASPO), **b** D-amino acid oxidase (DAAO), **c** serine racemase (SR), and **d** pLG72 proteins in the serum of controls (Ctrl, *n* = 12) and patients with non-treatment resistant (nTRS, *n* = 12), and treatment-resistant (TRS, *n* = 13) schizophrenia. Protein levels are expressed as extracted-ion chromatogram (XIC) intensity normalized to cytochrome C. **e** SR enzymatic activity expressed as μU/μL of serum. **a**–**e** Statistical analysis: Kruskall-Wallis test followed by Dunn’s test with Bonferroni’s correction for multiple comparison. *p ≤ 0.05, **p ≤ 0.01, ***p ≤ 0.0001. **f**–**m** Correlation between serum (**f**) DASPO and its substrate D-Asp, (**g**) DAAO and its substrate D-Ser, (**h**) SR and D-Asp or (**i**) D-Ser, **j** pLG72 and D-Asp or **k** D-Ser, and **l** pLG72 with DASPO or **m** DAAO in the serum of Ctrl, and patients with nTRS, and TRS schizophrenia. Statistical analysis: non-parametric Spearman correlation. *p ≤ 0.05, **p ≤ 0.01.

Serum levels of DASPO, DAAO, SR and pLG72 were measured in SCZ patients (*n* = 25), stratified into nTRS (*n* = 12) and TRS groups (*n* = 13), and non-psychiatric control subjects (*n* = 12) from “Federico II” University Hospital (Italy) (Fig. 2; Table 1, Suppl. Table 4), for whom D-AA serum levels had been previously analyzed^41^. No statistically significant differences were found in sex and age (Table 1). To assess the potential influence of antipsychotic treatment, a Spearman correlation analysis was performed between chlorpromazine (CPZ) equivalents^66^ and both enzyme and D-AA serum levels. No significant correlations emerged from this analysis (Suppl. Table 5), indicating that antipsychotic treatment did not significantly affect the measured parameters in our cohort, and was therefore not considered a confounding factor in subsequent analyses.

Non-parametric Kruskal-Wallis analysis revealed significant changes in serum levels of these proteins. In particular, serum DASPO and SR levels were increased in both nTRS and TRS groups compared with control subjects (DASPO: *p* = 0.0001, Kruskal-Wallis test; Ctrl vs nTRS *p* = 0.0001, Ctrl vs TRS *p* = 0.0069, Dunn’s test with Bonferroni’s correction for multiple comparisons. SR: *p* < 0.0001, Kruskal-Wallis test; Ctrl vs nTRS *p* < 0.0001, Ctrl vs TRS *p* = 0.0439; Dunn’s test with Bonferroni’s correction for multiple comparisons) (Fig. 2a, c; Suppl. Table 4; Suppl. Table 6). Interestingly, the increase in SR serum levels was higher in nTRS patients compared with TRS (*p* = 0.0349). On the other hand, DAAO and pLG72 levels were decreased in both groups of patients affected by SCZ (DAAO: *p* = 0.0005, Kruskal-Wallis test; Ctrl vs nTRS *p* = 0.0004, Ctrl vs TRS *p* = 0.0453; Dunn’s test with Bonferroni’s correction for multiple comparisons. pLG72: *p* = 0.0112, Kruskal-Wallis test; Ctrl vs nTRS *p* = 0.0449, Ctrl vs TRS *p* = 0.0181; Dunn’s test with Bonferroni’s correction for multiple comparisons) (Fig. 2b, d; Suppl. Table 4; Suppl. Table 6). We assessed catalase levels as a marker of peroxisomal integrity to rule out the possibility that peroxisomal damage could affect serum concentrations of peroxisomal DAAO and DASPO. Our findings show that catalase levels did not differ significantly between control subjects and patients with nTRS or TRS (Suppl. Figure 3a).

We then evaluated the enzymatic activity of DASPO and DAAO based on the detection of hydrogen peroxide, which showed no signal, pointing to a level lower than the assay sensitivity (LOD of 250 and 50 mU/mL for DAAO and DASPO, respectively, corresponding to a calculated 42 and 0.625 µg/mL of serum using as reference the specific activity of each pure recombinant enzyme). On the other hand, SR activity was detectable in serum samples and did not differ among SCZ patients and healthy controls (Fig. 2e).

We performed a statistical re-analysis of D-Ser and D-Asp serum levels in a subset of patients from our previous study^41^, restricted to the sub-cohort for whom enzymes have been quantified in the present study (Suppl. Table 6). Consistent with our previous findings^41^, we observed decreased D-Asp levels in both nTRS and TRS patients (Suppl. Table 6), as well as a selective reduction of D-Ser in TRS patients compared with the control group (Suppl. Table 6).

We then evaluated the correlation between serum levels of DASPO, DAAO, SR, and pLG72 proteins and D-Asp or D-Ser (Fig. 2f-k; Suppl. Table 7). Non-parametric Spearman’s correlation analysis did not show any significant association between DASPO, SR or pLG72 enzymes and D-Asp concentrations in both control subjects and SCZ groups, regardless of resistance to treatment (Fig. 2f, h, j; Suppl. Table 7). Concerning D-Ser, a significant positive correlation was observed exclusively in TRS patients between its serum concentration and pLG72 levels (r = 0.675, *p* = 0.013, Fig. 2k; Suppl. Table 7). Finally, we examined potential correlations between the flavooxidases DASPO and DAAO and their modulator, pLG72, across diagnostic groups (Fig. 2l, m). Intriguingly, in TRS patients, strong positive correlations were observed between pLG72 levels and both DAAO and DASPO. A similar correlation between DAAO and pLG72 was also detected in nTRS patients (Fig. 2l, m; Suppl. Table 7). These findings further support the involvement of the small primate-specific protein pLG72 in controlling the two flavooxidases.

### Increased serum DASPO and DAAO levels in ASD patient cohorts

We next measured the levels of proteins in the serum of paediatric ASD patients and control subjects recruited in two different Italian Hospitals (“Federico II” University Hospital and Istituto Giannina Gaslini: Ctrl, *n* = 17, ASD, *n* = 35; Table 1) for whom D-AA levels have been previously reported^41^.

**Fig. 3:**
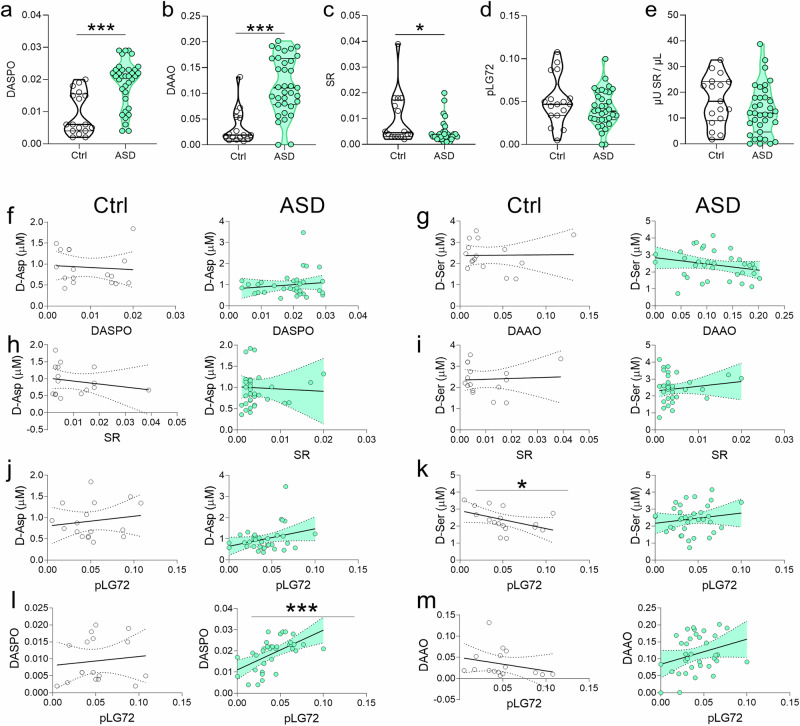
DAAO, DASPO and SR are dysregulated in the serum of patients with ASD. **a** D-aspartate oxidase (DASPO), **b** D-amino acid oxidase (DAAO), **c** serine racemase (SR), and **d** pLG72 proteins in the serum of paediatric controls (Ctrl, *n* = 17) and children affected by autism spectrum disorder (ASD, *n* = 35). Protein levels are expressed as extracted-ion chromatogram (XIC) intensity normalized to cytochrome C. **e** SR enzymatic activity expressed as μU/μL of serum. **a**–**e** Statistical analysis: Mann-Whitney test. *p ≤ 0.05, ***p ≤ 0.0001. **f**–**m** Correlation between serum (**f**) DASPO and its substrate D-Asp, **g** DAAO and its substrate D-Ser, **h** SR and D-Asp or (**i**) D-Ser, (**j**) pLG72 and D-Asp or (**k**) D-Ser, and (**l**) pLG72 with DASPO or (**m**) DAAO in the serum of Ctrl, and patients with ASD. Statistical analysis: non-parametric Spearman correlation. *p ≤ 0.05, ***p ≤ 0.0001.

In the ASD cohort (Fig. 3; Suppl. Table 8), we reported significantly increased DASPO and DAAO levels and decreased SR levels in ASD patients compared with control subjects (DASPO: *p* < 0.0001; DAAO: *p* < 0.0001, SR: *p* = 0.0126, Mann-Whitney’s U test; Fig. 3a-c; Suppl. Table 8; Suppl. Table 9). pLG72 levels as well as SR activity were unaltered between ASD and control patients (Fig. 3d,e; Suppl. Table 9). Since age [median (IQR) of years: Ctrl = 13.8 (8.6-15.3) vs ASD = 5.4 (3.9-9.1); *p* < 0.001], but not sex (χ^2^ = 0.683; *p* = 0.409), was found different between groups (Table 1), we also performed ANCOVA model considering the effect of age as possible confounder. This analysis confirmed higher serum DASPO and DAAO levels and lower SR content in ASD patients compared with controls (DASPO: *p* < 0.001; DAAO: *p* = 0.009; SR: *p* = 0.002, ANCOVA, Suppl. Table 9). Moreover, the absence of differences in serum catalase levels between ASD patients and controls indicates that the elevated DAAO and DASPO levels are not attributable to peroxisomal damage (Suppl. Figure 3b).

Consistent with previous findings^41^, D-Ser and D-Asp levels did not differ between ASD patients and healthy controls in the sub-cohort for which protein levels have been quantified (Suppl. Table 9). Non-parametric Spearman’s correlation analysis evidenced a negative correlation between D-Ser and pLG72 levels in the control group (*r* = -0.593, *p* = 0.014, Fig. 3k; Suppl. Table 10), while any significant association between other enzymes and D-AA concentrations in both control subjects and ASD patients was found (Fig. 3f-j; Suppl. Table 10). Notably, a positive correlation was again observed between DASPO or DAAO and pLG72 levels exclusively in ASD patients, with statistical relevance reached for DASPO (Fig. 3l; Suppl. Table 10). This suggests a mechanism through which pLG72 may avoid excessive D-Asp and D-Ser degradation, especially at increasing DASPO and DAAO levels, respectively.

Collectively, our results show a significant increase in serum DASPO and DAAO levels, and a decrease in SR, in ASD patients compared with control individuals, although the D-AA levels were unaffected.

## Discussion

SCZ and ASD are two complex neurodevelopmental disorders that have garnered increasing attention for their potential shared origins and pathophysiological mechanisms^67^. Both conditions typically emerge during early development and exhibit overlapping neurobiological features, including disruption in glutamatergic transmission, which may contribute to disrupting the structural and functional cortical-subcortical brain connectivity, in turn involved in functioning and cognition deficits^68,69^. Despite advances in research, significant challenges remain in the neurobiology of both psychiatric conditions.

In this multicentric study, we applied an MS-based approach to quantify the serum levels of key proteins involved in D-Asp and D-Ser metabolism, including DASPO, DAAO and SR, as well as pLG72, a protein involved in DASPO and DAAO enzymatic regulation. These levels were assessed in SCZ patients, stratified according to their response to antipsychotic treatment (nTRS and TRS patients), as well as in ASD patients, in comparison with their matched non-psychiatric controls.

Our results show that SR protein levels are increased in all SCZ patients, while the corresponding enzymatic activity does not change. Specifically, the reported SR overexpression may reflect a tissue-level compensatory mechanism aimed at counteracting the decreased serum D-Ser systemic levels observed in the same cohort of SCZ patients^41^, which likely mirror altered D-Ser levels in relevant tissues. However, a thorough understanding of the alteration in D-Ser metabolism in SCZ patients requires further investigation into the regulation of SR activity, as well as the biosynthesis of its precursor L-Ser (i.e. the phosphorylated pathway^49,70^), which needs to be further investigated.

Our findings also indicate altered D-Ser catabolism in SCZ, as both DAAO and pLG72 protein levels are significantly reduced in the serum of patients in comparison with the healthy subjects. The decrease in circulating DAAO levels may reflect a systemic compensatory mechanism aimed at limiting further D-Ser degradation. Since pLG72 regulates DAAO by promoting its inhibition and degradation, its reduced levels may follow the decrease in DAAO protein levels. Notably, human DAAO is characterized by a very low enzymatic activity, due to its low turnover rate and weak cofactor binding, and becomes more active in the presence of its substrate^11–13^. Therefore, the reduced serum D-Ser levels observed in SCZ patients, potentially mirroring tissue D-Ser decrease, may further attenuate DAAO activation, contributing to a broader decrease in D-Ser catabolism.

In contrast to what we observed for D-Ser catabolism, the decrease in serum D-Asp levels in both nTRS and TRS SCZ patients is accompanied by increased levels of DASPO. Although the relative levels of pLG72 are decreased in SCZ compared to healthy subjects, a statistically significant positive correlation is observed in TRS between DASPO and pLG72, the latter also acting as a negative modulator of this flavoenzyme by reducing both its activity and half-life^46,49^. This pattern may reflect an upregulation of D-Asp catabolism at the tissue level, potentially due to increased expression or activity of DASPO, with a concomitant relative rise in pLG72 as a regulatory response aimed at limiting excessive D-Asp degradation. In line with this interpretation, we have previously found reduced D-Asp levels in post-mortem cortical areas in two different SCZ patient cohorts, linked to increased DASPO mRNA expression or enzymatic activity^32,37,43^.

Despite our hypotheses, future studies are needed to assess whether serum D-Ser and D-Asp levels reliably reflect their metabolism in tissues. In this regard, it is important to emphasize that the enzymes regulating D-Ser and D-Asp metabolism primarily function in peripheral organs, such as kidney, liver, brain and others, rather than in the bloodstream, which likely reflects complex systemic regulatory mechanisms. Additionally, circulating levels of D-Ser and D-Asp are very low (in the low µM range or below), which complicates the interpretation of their systemic modulation.

Noteworthy, correlation analyses did not reveal consistent relationships between D-AA levels and their associated enzymes, suggesting that serum may not be a suitable biological matrix to capture these interactions. Additionally, the small sample size may have reduced the statistical power of these analyses, preventing any definitive conclusion. Moreover, we should remark that the correlation between serum D-AA levels and their regulatory enzymes is intrinsically complex. Indeed, systemic D-AA levels can be influenced by multiple factors, including dietary intake, metabolic rates and gut microbiota composition. In this regard, recent studies have indicated that the gut microbiota significantly contributes to the endogenous pool of D-AAs^71^, with bacterial enzymes actively involved in their metabolism^72,73^. Notably, gut dysbiosis, often triggered by inflammation or pharmacological treatments, has been widely reported in SCZ patients^74,75^ and may affect D-AA metabolism by impairing their systemic availability, thus adding further complexity to the interpretation of peripheral D-AA metabolism in neuropsychiatric conditions.

In ASD patients, we observed different alterations in the enzymes involved in D-Ser and D-Asp metabolism, although the levels of both D-AAs did not change significantly compared with healthy controls^41^. In particular, increased levels of both DAAO and DASPO were detected in ASD patient cohort. Moreover, a reduction in SR protein expression was observed. Taken together, these findings suggest that serum variations in DAAO, DASPO, and SR levels observed in ASD patient may not directly reflect circulating D-AA levels. Supporting this hypothesis, previous studies in rodent models of idiopathic ASD reported stable plasma and fecal D-AA levels despite cerebral alterations of both D-Asp and D-Ser, supporting the hypothesis that brain D-AAs-regulating enzymes may be dysfunctional and contribute to NMDAR-mediated dysfunction in ASD^43,44^.

Several studies have reported increased oxidative stress, lipid peroxidation, and impaired antioxidant defence systems – involving altered peroxisomal pathways – in patients with SCZ and ASD^76,77^. Given the established link between oxidative stress and neurodevelopmental disorders, peroxisomal dysfunction may contribute to the dysregulation of DAAO and DASPO, as both of which are localized within these organelles^11,22^. Future studies are needed to clarify whether these observed enzyme alterations in the bloodstream reflect oxidative stress-induced changes in peroxisomal metabolism.

A noteworthy finding of this study is the consistent positive correlation between pLG72 levels and those of DAAO and DASPO observed in both SCZ and ASD patient groups, as revealed by non-parametric Spearman analysis. This finding highlights the potential relevance of pLG72 protein as a shared regulatory hub in D-Ser and D-Asp catabolism through its modulation of DAAO and DASPO enzymes^2^ and indicates a convergent mechanism that reflects flavoenzyme alteration or compensatory efforts to limit their catabolic activity in neurodevelopmental disorders.

Regardless of their involvement in D-AA metabolism, the altered serum levels of DAAO, DASPO, SR and pLG72 in SCZ and ASD patients appear to represent condition-specific signatures. Importantly, unlike circulating D-AA levels, which can be influenced by external factors such as dietary intake, the expression levels of these proteins are less susceptible to such variability, thereby increasing their reliability as stable serum “fingerprints” for these psychiatric disorders.

We acknowledge some limitations in our study. First, as this is an observational study, we cannot draw causal conclusions about the relationship between amino acid variations and the levels of D-AA-regulating enzymes observed in ASD and SCZ patients. Second, we did not include drug-free SCZ patients in our research, which restricts the applicability of our biochemical findings to only those patients who are undergoing antipsychotic treatment. Third, all participants were of Caucasian origin; thus, the findings require validation in more diverse ethnic populations to ensure broader applicability. Finally, the relatively small number of cases and controls analyzed, and the inability to convert protein levels into absolute concentrations due to their low abundance, further represent a weakness of the present work. Despite these limitations, to our knowledge, this is the first report describing variations in the full set of enzymes and regulatory proteins primarily involved in D-AA metabolism. For this analysis, we developed and applied an optimized MS-based approach that offers high specificity for the targeted detection and relative quantification of these proteins. Moreover, the study was conducted in two major neuropsychiatric disorders, SCZ and ASD, through a multicenter design, thereby strengthening the robustness and generalizability of the findings.

In conclusion, our findings reveal differential yet partially overlapping alterations in DAAO, DASPO, SR and pLG72 protein levels in SCZ and ASD, suggesting condition-specific protein signatures with potential value as peripheral biomarkers. This initial evidence lays the underground for future studies aimed at validating the diagnostic utility of D-AA metabolism-related enzymes and elucidating their mechanistic roles in disease pathophysiology.

## Supplementary information

Supplementary Information

Supplementary Information

## Data Availability

Experimental and/or clinical data analyzed in this study are included as Supplementary Data.
