# Supplementary material for: Blood levels of D-aspartate oxidase, D-amino acid oxidase, serine racemase, and pLG72 are influenced by diagnoses of schizophrenia and autism spectrum disorder
Source: Schizophrenia (Heidelb). 2026 Apr 25;12(1):58. doi: 10.1038/s41537-026-00758-7 (PMC13324255; doi:10.1038/s41537-026-00758-7)
Supplement: Supplementary file 1 — Supplementary Information [file 41537_2026_758_MOESM1_ESM.docx]

*Supplementary information for*

**Blood levels of D-aspartate oxidase, D-amino acid oxidase, serine racemase, and pLG72 are influenced by diagnoses of schizophrenia and autism spectrum disorder.**

Elisa Maffioli^1,#^, Francesco Errico^2,3,#^, Zoraide Motta^4^, Raffaella di Vito^3,5^, Joshua Grana^1^, Elisa De Grandis^6,7^, Silvia Boeri^6,7^, Claudio Bruno^6,8^, Maria Pia Riccio^9^, Felice Iasevoli^10^, Michele Di Maio^3^, Tommaso Nuzzo^3,5^, Carmela Bravaccio^11^, Sveva Bagnasco^3,12^, Monica Gelzo^3,13^, Giuseppe Castaldo^3,13^, Andrea de Bartolomeis^10^, Armando Negri^1^, Loredano Pollegioni^4^, Gabriella Tedeschi^1,^*, Alessandro Usiello^3,5,^*

^1^DIVAS, Department of Veterinary Medicine and Animal Sciences, University of Milano, Milan, Italy;

^2^Department of Agricultural Science, University of Naples “Federico II”, Portici, Italy;

^3^CEINGE Biotecnologie Avanzate “Franco Salvatore”, Naples, Italy;

^4^“The Protein Factory 2.0”, Dipartimento di Biotecnologie e Scienze della Vita, Università degli Studi dell’Insubria, Varese, Italy;

^5^ Department of Environmental, Biological and Pharmaceutical Sciences and Technologies, Università degli Studi della Campania “Luigi Vanvitelli”, Caserta, Italy

^6^Department of Neuroscience, Rehabilitation, Ophthalmology, Genetics, Maternal, and Child Health – DINOGMI, University of Genoa, Genoa, Italy;

^7^ Child Neuropsychiatry Unit, IRCCS Istituto Giannina Gaslini, Genoa, Italy

^8^Center of Translational and Experimental Myology, Istituto di Ricovero e Cura a Carattere Scientifico (IRCCS) Istituto Giannina Gaslini, Genoa, Italy;

^9^Department of Maternal and Child Health, Unità Operativa semplice di Dipartimento (UOSD) of Child and Adolescent Psychiatry, Azienda Ospedaliera Universitaria (AOU) Federico II, Naples, Italy;

^10^Section of Psychiatry, Laboratory of Translational and Molecular Psychiatry and Unit of Treatment-Resistant Psychosis, Department of Neuroscience, Reproductive Sciences and Odontostomatology, University Medical School of Naples “Federico II”, Naples, Italy;

^11^Department of Medical and Translational Sciences, Child Neuropsychiatry, Federico II University, Napoli, Italy;

^12^Dipartimento di Medicina di Precisione in Area Medica, Chirurgica e Critica, Università di Palermo, Palermo, Italy

^13^Department of Molecular Medicine and Medical Biotechnologies, Federico II University of Naples, Naples, Italy

^#^These authors have contributed equally to this work.

*Correspondence:

Gabriella Tedeschi, email: [gabriella.tedeschi@unimi.it](mailto:gabriella.tedeschi@unimi.it), phone: +39025033454;

Alessandro Usiello, email: [usiello@ceinge.unina.it](mailto:usiello@ceinge.unina.it), phone: +390813737899.

**Keywords:** DASPO, DAAO, SR, pLG72, neuropsychiatric disorders, PRM, D-amino acids.


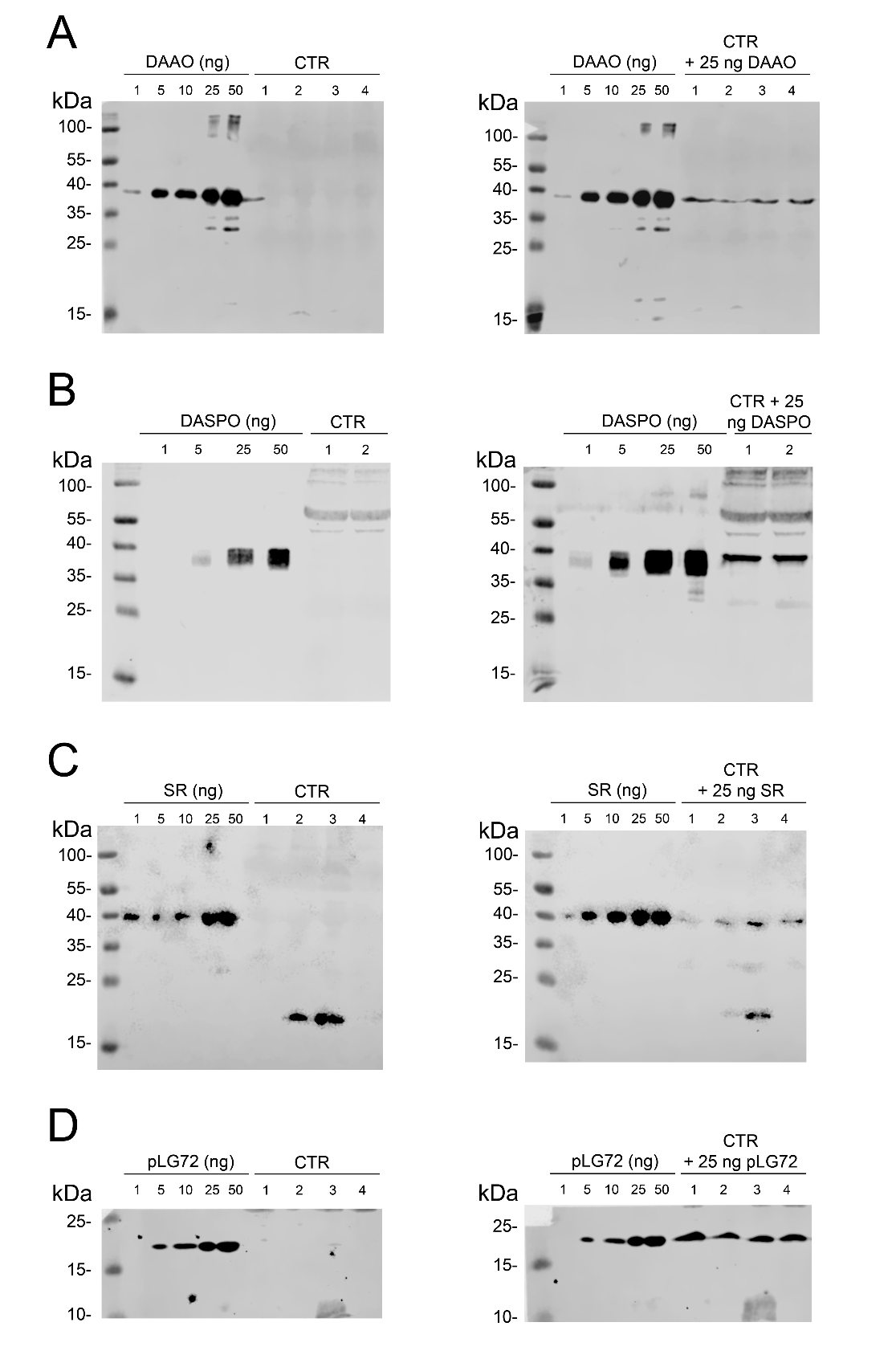


**Suppl. Figure 1.** Western blot analyses did not identify the proteins related to D-Ser and D-Asp metabolism in human blood: detection limit was 1 ng for DAAO (A) and SR (C), and 5 ng for DASPO (B) and pLG72 (D). Left: 40 μL of serum samples; right: 40 μL of serum samples added of 25 ng of recombinant purified proteins. Lanes 1-4: control samples.


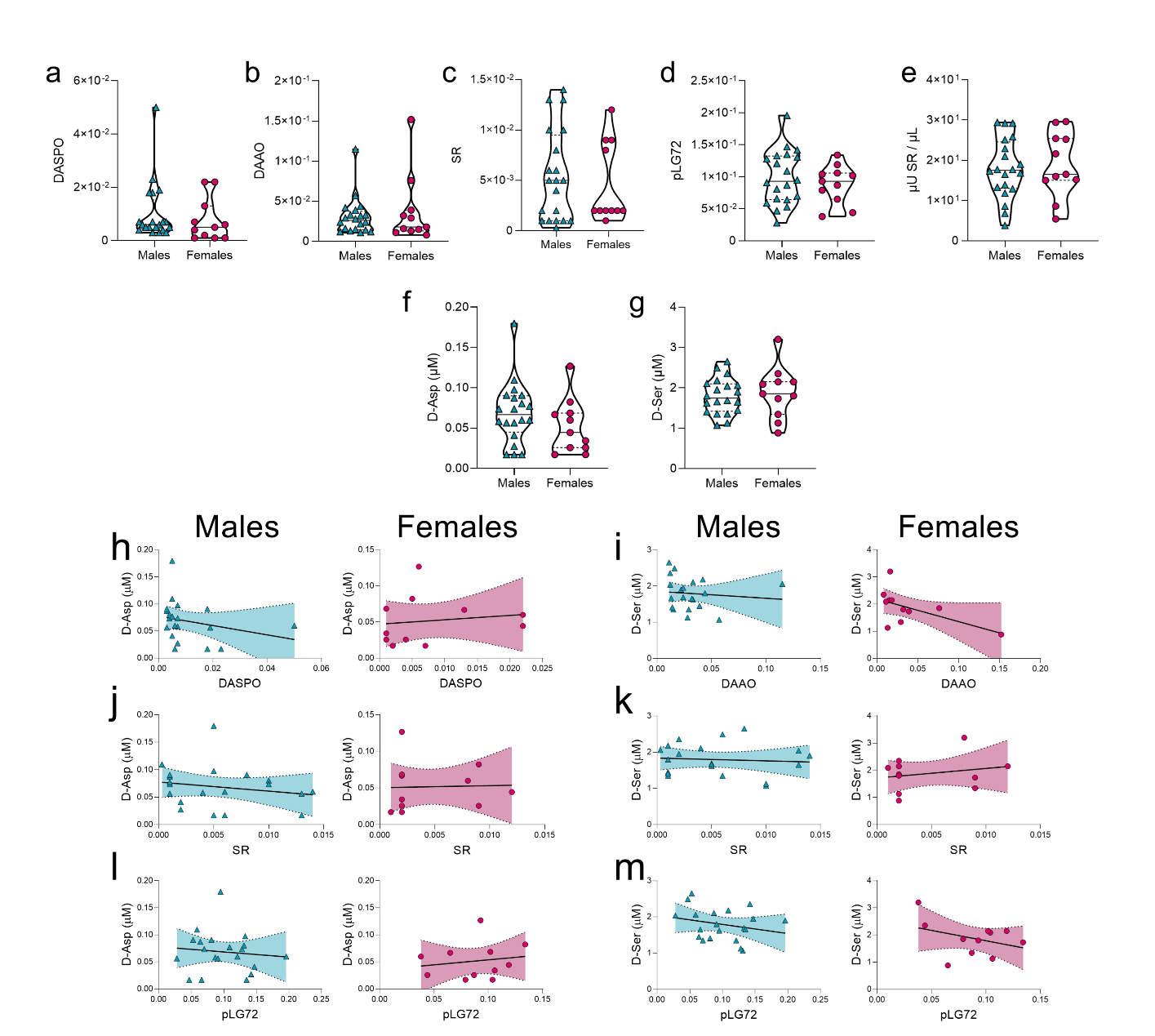


**Suppl. Figure 2**. **Serum levels of D-amino acids, D-amino acid metabolizing enzymes, and SR activity are comparable between male and female healthy subjects.** (a) D-aspartate oxidase (DASPO), (b) D-amino acid oxidase (DAAO), (c) serine racemase (SR), and (d) pLG72 protein levels in the serum of male (n=20) and female (n=12) healthy subjects. Protein levels are expressed as extracted-ion chromatogram (XIC) intensity normalized to cytochrome C. (e) SR activity expressed as μU/μL of serum. (f) D-Asp (μM) and (g) D-Ser (μM) blood serum concentration in male and female healthy subjects. (a-g) Statistical analysis: Mann-Whitney test. Correlation between serum (h) DASPO and its substrate D-Asp, (i) DAAO and its substrate D-Ser, (j) SR and D-Asp or (k) D-Ser, and (l) pLG72 and D-Asp or (m) D-Ser in female and male healthy subjects. (h-m) Statistical analysis: non-parametric Spearman correlation**.**

**
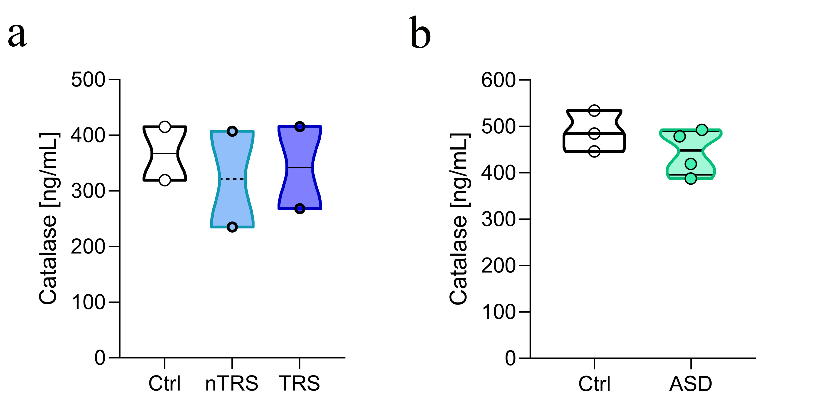
**

**Suppl. Figure 3.** Serum levels of catalase. (a) Comparison among controls (Ctrl, n = 2) and SCZ patients divided in non-treatment-resistant (nTRS n = 2), treatment-resistant (TRS, n =2) groups. (b) Comparison between controls (Ctrl, n = 3) and autistic patients (ASD, n = 4). Catalase levels are expressed in ng/mL serum. Graph report the mean of duplicate measurements for each pooled sample. No statistically significant differences were found between the groups (non-parametric Kruskal-Wallis test, followed by post-hoc Dunn’s test with Bonferroni's correction for SCZ and Mann-Whitney test for ASD) using GraphPad Prism 9.0.
